# Davis’ Clinical Lectures

**Published:** 1874-12

**Authors:** 


Clinical Lectures on Various Important Diseases; being a collection of the clinical lectures delivered in the medical wards of Mercy Hospital, Chicago. By Nathan S. Davis, A.M., M.D., Professor of Principles and Practice of Medicine, and Clinical Medicine, in Chicago Medical College. Edited by Frank M. Davis, M.D. Second edition. Philadelphia: Henry C. Lea, 1874; 12mo.; cloth, pp. 283. Price not stated.

This volume embodies a series of clinical lectures originally reported for the *Chicago Medical Examiner*. The subjects treated are: Continued fever; periodical fever; rheumatic fever; scarlatina and rubeola; respiratory affections; pulmonary tuberculosis; diseases of the alimentary tract; intestinal and uterine irritation; summer complaints of children; dropsy; cardiac disease; neuralgia; nervous affections; affections of the brain; cerebro-spinal diseases; cutaneous diseases; mania-a-potu and chronic diseases of the brain; and pneumonia.

---

PROCEEDINGS OF THE MEDICAL ASSOCIATION OF THE STATE OF ARKANSAS. Fifth annual session, held at Little Rock, October 20th and 21st, 1874. Octavo pamphlet, pp. 43.

---

VARICOCELE AND ITS RADICAL CURE. By Octavius A. White, A.M., M.D., of New York. Reprinted from the *New York Medical Journal*, July, 1873. Octavo pamphlet, pp. 13.

---

THE LEGAL RELATIONS OF EMOTIONAL INSANITY. By E. Lloyd Howard, M.D., Baltimore, Md. Extracted from the *Transactions of the American Medical Association.* Octavo pamphlet, pp. 22.

